# Influenza A Virus Antibodies with Antibody-Dependent Cellular Cytotoxicity Function

**DOI:** 10.3390/v12030276

**Published:** 2020-03-01

**Authors:** Rongyuan Gao, Zizhang Sheng, Chithra C. Sreenivasan, Dan Wang, Feng Li

**Affiliations:** 1 Department of Biology and Microbiology, South Dakota State University, Brookings, SD 57007, USA; rongyuan.gao@sdstate.edu (R.G.); chithra.sreenivasan@sdstate.edu (C.C.S.); 2 Zuckerman Institute, Columbia University, New York, NY 10027, USA; zs2248@columbia.edu; 3 BioSNTR, Brookings, SD 57007, USA

**Keywords:** influenza, non-neutralizing monoclonal antibody, antibody-dependent cellular cytotoxicity (ADCC), Fc effector activities

## Abstract

Influenza causes millions of cases of hospitalizations annually and remains a public health concern on a global scale. Vaccines are developed and have proven to be the most effective countermeasures against influenza infection. Their efficacy has been largely evaluated by hemagglutinin inhibition (HI) titers exhibited by vaccine-induced neutralizing antibodies, which correlate fairly well with vaccine-conferred protection. Contrarily, non-neutralizing antibodies and their therapeutic potential are less well defined, yet, recent advances in anti-influenza antibody research indicate that non-neutralizing Fc-effector activities, especially antibody-dependent cellular cytotoxicity (ADCC), also serve as a critical mechanism in antibody-mediated anti-influenza host response. Monoclonal antibodies (mAbs) with Fc-effector activities have the potential for prophylactic and therapeutic treatment of influenza infection. Inducing mAbs mediated Fc-effector functions could be a complementary or alternative approach to the existing neutralizing antibody-based prevention and therapy. This review mainly discusses recent advances in Fc-effector functions, especially ADCC and their potential role in influenza countermeasures. Considering the complexity of anti-influenza approaches, future vaccines may need a cocktail of immunogens in order to elicit antibodies with broad-spectrum protection via multiple protective mechanisms.

## 1. Introduction

Influenza viruses cause severe respiratory illness, leading to 290,000–650,000 deaths annually worldwide as estimated by the World Health Organization (WHO) [1]. Influenza viruses consist of four known genera, A, B, C, and D, which belong to the family *Orthomyxoviridae* [2]. Of these four genera, influenza A virus causes the greatest mortality and is the most common cause of both seasonal and pandemic influenza outbreaks. Influenza B virus can cause seasonal influenza while the influenza C virus can infect children with mild respiratory symptoms. Little is known about the impact of recently discovered influenza D virus on human health [3,4]. 

**Figure 1:**
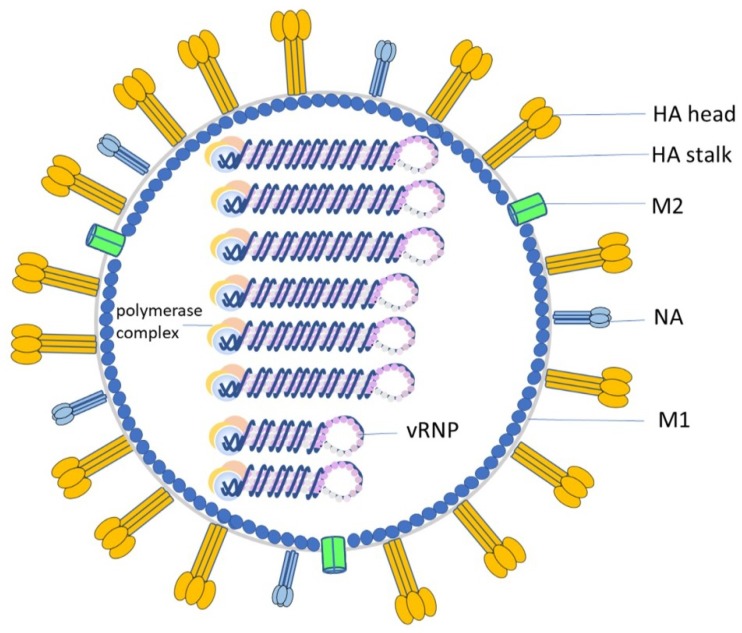
Schematic view of influenza virion. Hemagglutinin (HA), neuraminidase (NA) and matrix protein 2 (M2) are the proteins expressed on virus membrane. Trimeric HA protein consists of HA head and HA stalk. Viral ribonucleoprotein (vRNP) is composed of vRNA and nucleoprotein (NP, light purple). Viral polymerase complex includes polymerase basic proteins 1 (PB1, blue), 2 (PB2, tan) and acidic protein A (PA, light yellow). The matrix protein (M1) is a multi-functional protein involved in influenza virion assembly and infection.

Hemagglutinin (HA) and neuraminidase (NA) are the two primary viral surface glycoproteins (Figure 1) involved in critical steps of the influenza life cycle. The trimeric HA protein is comprised of two domains: the head domain and the stalk domain. HA head domain contains the receptor binding site (RBS) that binds to sialic acids (SAs) on the susceptible cells to initiate the virus’ replication cycle. After the virus is endocytosed, the fusion peptide in the HA stalk was exposed to mediate the membrane fusion towards releasing viral ribonucleoprotein (RNP) complex into the cytoplasm and subsequently to the nucleus of infected cells [2]. After the newly assembled influenza virus buds from the infected cell, HA on the virion still interacts with the SA receptors on the host cell membrane. The tetrameric NA spike functions to release the viral progeny through cleaving α-ketosidic linkage between the SA and an adjacent sugar residue [5].

The HA and NA proteins are also highly immunogenic and antibodies targeting both glycoproteins can be isolated after natural infection or vaccination. Through binding to viral surface proteins HA and NA, antibodies can block the essential steps in the virus replication cycle, thereby limiting the spread of infection. Due to the host immune pressure and error-prone RNA polymerase, HA and NA are very plastic and display difference in antigenic properties. According to the antigenic difference, influenza A virus can divide into 18 HA (H1–H18) and 11 NA (N1–N11) subtypes [6]. According to the Weekly U.S. Influenza Surveillance Report released by CDC, H1N1(pdm09) and B/Victoria lineage viruses are equally dominant and responsible for the majority of death cases during the 2019–2020 influenza season [7].

Vaccination is the best countermeasure to prevent and control influenza [8]. Live attenuated, inactivated and recombinant HA vaccines are the three types of licensed seasonal influenza vaccines [9]. These vaccines conferred considerable protection in combating influenza by inducing antibodies that target HA. However, their efficacy can be significantly reduced when novel viruses emerge, or when there is a mismatch between the vaccine strain and the circulating influenza strain [8]. Therefore, the ultimate goal is to develop a universal vaccine that could confer long-lasting protection against multiple influenza strains, including the drifted seasonal influenza viruses and antigenically distinct viruses. Several approaches are employed to achieve that goal, such as stalk-based immunogen [10], chimeric HA immunogen strategies [11] and computationally optimized broadly reactive antigen (COBRA)-based vaccines [12,13]. Elicitation of antibodies exhibiting ADCC activities also contributes to the design of universal vaccines, which are thought to confer broad-spectrum protection [14].

Previous antibody protective efficiency was measured by their capability to prevent HA binding via neutralization assay and hemagglutination inhibition assay [14,15], antibodies without these functions were less well defined. However, increasing evidence suggests that non-neutralizing antibodies (nnAbs) can confer protection via multiple mechanisms without disturbing virus entry or membrane fusion, such as activating complement, increasing phagocytosis, targeting internal viral proteins and eliciting fragment crystallizable (Fc)-effector functions [16,17,18,19]. Unlike neutralizing antibodies, nnAbs do not bind to the RBS in the HA head and fail to inhibit hemagglutination. Therefore, these antibodies demonstrate no detectable *in vitro* hemagglutination inhibition (HI) titer or neutralizing activity commonly considered as the benchmark standard of vaccine efficacy. Nonetheless, recent studies have validated that nnAbs can also contribute to the vaccine-elicited protection against influenza virus infection *in vivo* [20,21]. The protective mechanisms employed by nnAbs depend on the engagement with effector cells and consist of antibody-dependent cellular cytotoxicity (ADCC), complement-dependent cytotoxicity (CDC), and antibody-dependent cell-mediated phagocytosis (ADCP). ADCP also assists in the elimination of virus-infected cells and protects against influenza virus infection in a mouse model [22]. Importantly, these nnAbs can be induced by vaccination in humans [18,20,23]. Jegaskanda et al. showed that ADCC-mediated antibodies (ADCC-Abs) can be induced from monovalent inactivated influenza A(H1N1) virus vaccine, high titers of ADCC-Abs can contribute to lower virus replication and reduced clinical symptoms [20]. Zhong and colleagues demonstrated that both ADCC-Abs and neutralizing antibodies can be elicited by influenza vaccination [23].

The mechanism of the antibody-triggered Fc-effector functions is well understood. The antibody Fc region interacts with the FcγRIIIa (CD16a) on the surface of the Fc receptor-bearing cells, primarily natural killer cells (NK cells). After the antibodies bind to their target viral proteins, the combined interactions activate these effector cells. The C-terminal immunoreceptor tyrosine-based activation motif (ITAM) in effector cells is subsequently activated to stimulate the release of granzyme B and perforin by activating signal-transduction molecules and various pathways, such as calcium-dependent pathways and mitogen-activated protein kinase pathway (originally known as extracellular signal-regulated kinases pathway) [24,25]. Details of the signaling pathways initiated by Fc-Fc receptors (FcRs) have been extensively reviewed by Nimmerjahn et al [26]. Perforins can produce pores in the virus-infected cell surface, allowing the entry of granzyme B that cleaves death substrate to initiate apoptosis [27]. Together, these facilitate the elimination of influenza virus-infected cells [28]. This Fc-receptor binding activity was also confirmed to confer protection in animal models in the absence of neutralizing antibodies [29].

Multiple parameters impact the potency of Fc-effector functions. FcRs expression pattern in these cells affects the potency of Fc-effector functions. FcRs are divided into activating and inhibitory receptors based on whether the receptor-mediated signal activates the ITAM or the opposing immunoreceptor tyrosine-based inhibitory motif (ITIM). The magnitude of NK cell responses critically relies on the integration of signals transduced by ITAM and ITIM [30]. Depending on the antibody isotype, these binding receptors can differ. FcγR, FcεR, and FcαR can recognize Fc region of IgG, IgE, and IgA, respectively [31]. FcγRs are the most well-studied FcRs. Different FcγRs can vary significantly from antibody binding affinity to ADCC induction [32]. In human FcRs, FcγRI(CD64), FcγRIIa(CD32a), FcγRIIc(CD32c), and FcγRIIIa(CD16a) are activating receptors, while FcγRIIb (CD32b) is the inhibitory receptor [33]. It is worth noting that no inhibitory receptor is present on NK cells’ surface, only the activating receptor FcγRIIIa is expressed [26]. Without the intervention of FcγRIIb as an inhibitory receptor to negatively regulate cell activation, NK cells serve as the predominant cells that can mediate potent ADCC responses *in vivo* [28]. 

Although mouse FcγRs and their counterparts in humans are named similarly, they may differ substantially in their expression pattern, antibody subclass binding affinities and *in vivo* dominant FcγRs [29,33]. Some FcγRs, such as FcγRIIa, FcγRIIc, and FcγRIIIb, are expressed in human immune cells but not in mice cells [34] and FcγRIV is expressed in mice immune cells but not in human cells [35]. Therefore, a transgenic mouse model consistently expressing human FcRs is needed when investigating human antibody-mediated Fc-effector functions and the human FcγR contribution to antiviral response. Smith et al. generated a mouse model without any murine FcγRs and human FcγRs genes were inserted into the mouse genome to express functional human FcγRs [36]. These human FcγRs were validated to mediate the cytotoxic effects of human IgG antibodies, providing a valuable platform for therapeutic IgG antibodies studies [29].

Most therapeutic antibodies in development are IgG [37]. IgG subclasses (IgG1, IgG2, IgG3, and IgG4 in humans) exhibited different binding affinities to FcγRs as they differ in primary sequence and the associated structure. Of the four IgG subclasses, IgG1, IgG2, and IgG4 are used clinically for therapeutic purposes [38], while the extensive application of IgG3 may be hindered by the its short serum half-life [39,40]. Both IgG1 and IgG3 can mediate potent ADCC activity, while IgG2 and IgG4 have a poor ADCC function [41].

Lastly, antibody glycosylation is essential for maintaining antibody structural conformation which is directly related to the potency of Fc-effector functions [37,42]. Many factors can affect glycosylation patterns, which include age, pregnancy and virus infection [43,44,45]. However, the detailed mechanism of how antibody glycosylation impact Fc-effector functions remains unclear. It has been demonstrated that the reduced content of fucose can enhance ADCC activity [46]. Various approaches, such as alpha-(1,6)-fucosyltransferase gene knockout, have been developed to manipulate antibody fucosylation to optimize ADCC activity [47]. Glyco-engineering and protein engineering have emerged as the two main strategies to harness ADCC activity [48].

Antibodies mediating Fc-effector activities were isolated and characterized in numerous studies, which suggest that these antibodies can play a critical role in tumor clearance [25] and immunity to multiple pathogens such as human immunodeficiency virus (HIV) [49,50], Ebola [51], dengue virus (DENV) [52], West Nile virus (WNV) [53] and influenza virus [16,17]. HIV infection remains a health issue on a global scale and vaccine candidates were developed to address this deteriorating issue. Currently, only one HIV vaccine, RV144, shows modest efficacy (31% efficacy) and this efficacy is not associated with neutralizing antibodies but with potent ADCC-Abs [54,55,56,57,58]. 

## 2. Antibodies Induce ADCC Activities through Diverse Innate Effector Cells

As Fc-FcγR interactions are required to induce ADCC, effector cells must express FcγRs on membrane surface to facilitate Fc binding. The NK cell is not the only cell type that has the FcγRIIIa on the membrane, other Fc receptor-bearing cells, like neutrophils, monocytes/macrophages, granulocytes, and dendritic cells (DCs) may also play a crucial role in mediating ADCC or ADCP for viral infected cell clearance [26,59,60]. Broadly neutralizing mAbs targeting HA-stalk can induce ADCP by neutrophils, which indicate the important role of neutrophils in viral clearance [61,62]. Ackerman et al. described a high-throughput flow cytometry-based assay to measure the phagocytic activity by using a monocytic cell line [63]. In addition, Horner et al. reported the critical role of polymorphonuclear granulocyte in mediating ADCC activity in combating malignant diseases [64].

## 3. *In Vitro* Assays to Assess Antibody-Induced ADCC Activities

Since the discovery of the ADCC phenomenon in 1965 by Erna Moeller [65], multiple assays have been developed to measure ADCC activity for different purposes and are extensively employed in vaccine study and drug discovery. To measure ADCC activities *in vitro*, virus-infected target cells, antibodies, and effector cells are needed. The various assays differ in the type of effector cells used or the way to measure the cytotoxicity, which have contributed to high variability among the assays. Considering the complex mechanism of ADCC, it remains questionable which assays are the most physiologically relevant to predict the *in vivo* efficacy [56,66].

### 3.1. ^51^Cr Release Assay

ADCC activity is measured indirectly by the release of ^51^Chromium(^51^Cr) following lysis of virus-infected target cells in early studies [67,68,69]. In this assay, the target cells are labeled with ^51^Cr and subsequently incubated with the appropriate antibody. The quantity of ^51^Cr released from the lysed cells is detected by a gamma counter as a correlate of ADCC activity [69]. However, the biohazard problem of radioisotopes plus the test’s low sensitivity restricts the wide application of this assay. For these reasons, nonradioactive assays with better sensitivity are later developed to assess ADCC activities [70].

### 3.2. Target Cell Killing Assay

Flow cytometry-based ADCC assays are developed to measure ADCC activity by using different fluorescent dyes to differentiate living and dead cells [70,71]. In this assay, PKH-67 is used to label both live and dead target cells while dead target cells and effector cells are labeled with 7-amino actinomycin D (7-AAD). Instead of measuring reporter molecules released from target cells, this assay directly measures the lysed target cells. The quantification of lysed target cells is determined by measuring the double fluorescent dyes positive cells by flow cytometry. Target cell death in the absence of antibody is used as a background control. In 1990, Radosevic et al. reported their ADCC assay using different fluorochromes for cell staining with similar principles [72]. Compared to other ADCC measurement assays, this flow cytometry-based assay has a number of advantages such as simplicity, sensitivity, and accessibility, which gains it popularity in many laboratories [72].

### 3.3. NK Cell Activation Assay

NK cell activation assay is employed extensively to evaluate ADCC activities *in vitro* due to its high-throughput and precision [16,29,57,73,74]. In this flow cytometry-based assay, recombinant proteins or virus-infected cells are coated in plates followed by the addition of antibody and peripheral blood mononuclear cells (or NK cell lines). Lysosomal-associated membrane protein-1 (LAMP-1 or CD107a) is a cell marker expressed on the NK surface and is employed in this assay to identify NK cells’ functional activities as the expression level of CD107a is highly correlated with cytokine secretion from activated NK cells [75,76]. Therefore, the potency of ADCC activities can be determined indirectly by measuring the activation marker CD107a expression on the NK cell surface. This assay has been widely used to assess ADCC activities evoked by antibodies against the influenza virus and HIV [77,78].

### 3.4. ADCC Reporter Bioassay

This bioluminescent reporter assay is designed to determine ADCC activities by the measurement of firefly luciferase expression in effector cells [79]. A modified Jurkat stable cell line expressing FcγRIIIa is used as effector cells and expresses firefly luciferase when activated by nuclear factor of activated T cells (NFAT)-response element during the time when FcγRs are bound by Fc portion of the antibody. This cell line is developed in a simple thaw-and-use format which reduces the high inter-assay variability and greatly increases the throughput. This commercially available assay has been employed to quantify ADCC activities against influenza infections [17,80,81,82,83,84]. 

## 4. ADCC Induced by anti-HA Antibodies

### 4.1. HA Stalk-Targeting mAbs Can Mediate Robust ADCC Activities

The HA stalk region, in contrast to the HA head region, is less immunogenic. The antigenic subdominance of HA stalk region may be due to the steric shielding of HA head domain, making it less accessible to the humoral immunity [85]. Therefore, antibodies against the stalk region are not readily stimulated and detected in natural infection of humans. Nevertheless, low levels of stalk-specific B cells are detected in influenza patients [86]. Furthermore, anti-stalk antibodies with broad neutralizing activity have been isolated and characterized [87,88,89,90,91,92]. Since the stalk domain is more conserved than the head domain, these antibodies exhibit broad-spectrum binding and are capable of conferring heterosubtypic protection by interfering with the membrane fusion. HA stalk antibodies were shown to neutralize subtypes in the same group or cross both group 1 and group 2 [93,94]. Ekiert et al. reported that HA stalk-binding human antibody, CR6261, neutralizes multiple influenza subtypes from group 1 [95]. Corti et al. found that one antibody generated from single human plasma targeting fusion sub-domain of the HA stalk could bind and neutralize influenza A viruses from both group 1 and group 2 [94]. Dreyfus et al. reported an HA stalk-binding antibody, CR9114, broad binding to influenza A and B HAs and the antibody was able to confer protection against not only influenza A virus infection, but also influenza B virus infection in mice [89]. It should be noted that some antibodies targeting the stalk region are not highly potent when compared to those against the head region. This deficiency should be addressed in future studies.

Through inhibiting conformational changes essential to the membrane fusion, HA stalk-binding antibodies can disarm influenza virus infectivity [96]. Headless HA and chimeric HA are the two strategies to elicit HA stalk-reactive antibodies [86]. These stalk-reactive antibodies exhibit higher protective potency *in vivo* than *in vitro* since these antibodies may be capable of engaging FcγR-bearing cells to confer Fc-dependent protection [97]. Vries et al. showed that anti-H1 stalk ADCC-antibody can be detected after the immunization with a chimeric HA consisting of an H1 stalk domain and an irrelevant head domain [21]. The incorporation of two anti-viral mechanisms has attracted substantial attention in influenza vaccine design. Those HA stalk vaccine candidates were tested in human clinical trials [98,99]. Unfortunately, clinical outcomes of HA stalk-based vaccines are not encouraging so further clinical study was recently discontinued, which emphasizes a critical need to discover novel vaccines that are capable of inducing potent cross-subtype protective antibodies with ADCC activity (https://www.fiercebiotech.com/biotech/gsk-dumps-universal-flu-vaccine-after-interim-data-readout). 

### 4.2. HI-Negative Head-Targeting mAbs Can Mediate Robust ADCC Activities

**Figure 2:**
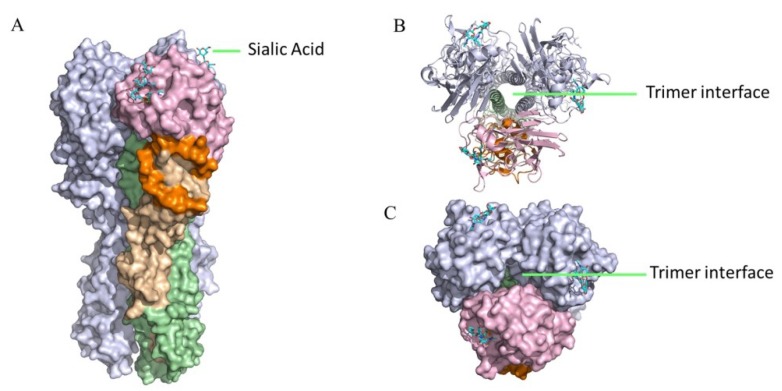
Antibody-dependent cellular cytotoxicity (ADCC)-epitopes in HA. Antibodies targeting HA stalk, as well as HA head including esterase domain and trimer interface, can induce potent ADCC *in vitro* and elicit broad-spectrum protection *in vivo*. The HA structure is generated using PyMOL. A/California 04/2009 HA (PDB code: 3UBE) is used as a representative HA structure to show the location of ADCC-epitopes. Influenza HA is a trimer with each monomer comprised of HA1(wheat), vestigial esterase domain (orange) and receptor binding domain (pink), and HA2 (palegreen). The remaining two monomers are shown in light blue. Sialic acid is colored cyan. Side view of the HA trimer surface is shown in (**A**), while the top view of the HA trimer interface is shown in the cartoon (**B**) and surface (**C**) modes. Besides, there are ADCC-Abs targeting HA head with unknown epitopes [100].

The anti-stalk antibodies can induce ADCC activities through engaging FcγRs on NK cells, but the role of anti-HA head mAbs in inducing ADCC remains unclear. Dilillo et al. used an FcγR knock-out mice model to show that only anti-HA stalk mAbs can bind to FcγRs and induce ADCC potently whereas anti-HA head mAbs inefficiently induce ADCC activity due to poor engagement of FcγRs on the NK cell surface [29]. Other research groups also reported on these findings independently [73,83]. Interestingly, the examination of anti-HA head mAbs for mediating ADCC in different studies achieved distinct results. Recent studies suggested that some HA-head reactive antibodies (HI-negative) could also engage FcγRIIIa on NK cells and mediate ADCC activity (Figure 2). 

Firstly, epitopes in the vestigial esterase domain on HA head have also shown some promise as ADCC-Ab binding targets (Figure 2A). Vestigial esterase domain-binding antibodies were reported to function mainly by blocking viral egress similar to NA inhibitors [89,101]. Recent studies indicated that vestigial esterase domain-binding antibodies also elicit ADCC that may help to protect against viral infection [102]. Bangaru and colleagues found a monoclonal antibody, H3v-47, that not only can neutralize diverse H3N2 viruses and block progeny viruses release, but also induce potent ADCC activity, suggesting that Fc-effector functions are critical for both HA head and stalk targeting antibodies [102]. Interestingly, these researchers found that this multifunctional antibody can recognize a novel epitope that spans the vestigial esterase and receptor-binding subdomains. Similar Fc-mediated ADCC activity was observed in mAb 46B8 that targets the vestigial esterase domain of HA head of the influenza B virus [74]. In addition, the vestigial esterase domain is highly conserved, indicating a potential role in universal vaccine design.

Secondly, antibodies targeting epitopes in the trimer interface may also initiate Fc-effector activities. Watanabe et al. reported that antibody (S5V2-29) binding to a conserved epitope at the HA head trimer interface can also confer potent protection *in vivo* by activating FcγR-dependent effector activities [103]. Bangaru et al. discovered a broadly protective antibody, FluA-20, which recognized a well-conserved epitope occluded in the trimer interface and also exhibited robust Fc-mediated ADCC *in vitro* [104]. However, the ADCC may not be essential to the protective role in mice. Historically, it is believed that the trimeric HA protein is so stable that the trimer interface may not be exposed to the host immune system and is likely unreachable by antibody but, contrarily, the epitopes identified by these researchers suggest that the trimer interface may be accessible, at least partially. As such, the trimer interface can be further explored as a potential target for vaccine and therapeutics.

Thirdly, some HA-head reactive antibodies with undefined epitopes were reported to confer protection *in vivo* via Fc-effector functions. DiLillio et al. demonstrated that the broadly neutralizing HA head-reactive antibodies, 4G05 and 1F05, can induce protection *in vivo* through Fc-FcγR interactions whereas strain-specific HA head reactive antibodies confer protection in an Fc-FcγR interactions-independent manner *in vivo* [100]. In addition, anti-H4 monoclonal antibodies binding to the HA head showed high cross-reactivity to avian and mammalian H4 HAs and protected mice from lethal H4N6 challenges through ADCC activity [81]. Two highly conserved epitopes, designated E1 and E2, located in HA head of a pandemic H1N1 virus, were reported to be recognized by ADCC-Ab [70]. The discovery of HA head reactive antibodies and conserved epitopes may assist the future design of universal vaccines and therapeutics.

### 4.3. HI-Positive Head-Targeting mAbs Cannot Mediate Robust ADCC Activities

**Figure 3:**
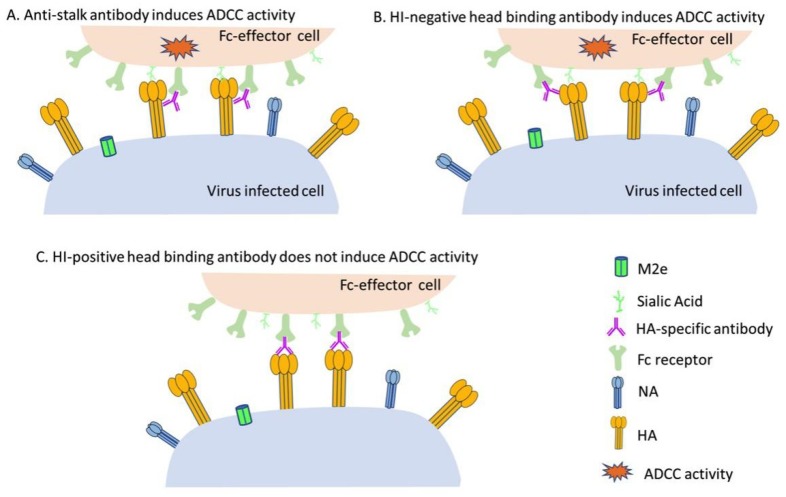
Two synapses between effector cell and virus-infected cell are essential for robust ADCC activities. The interaction between Fc and FcγRIIIa severs as the first synapse and the interaction between viral HA head and the sialic acid on effector cell form the second synapse, which are indispensable for inducing potent ADCC activities (**A**,**B**). The HI-positive antibody may interfere with the interaction between viral HA head and the sialic acid on effector cell, leading to weak ADCC activity (**C**). HI assay was extensively performed to determine the effects of antibodies in blocking the receptor engagement and replication of the viruses. However, this evaluation may be challenged by some emerging influenza viruses that lack the ability to agglutinate red blood cells of various animal origins [107]. In this scenario, the cell-based virus-neutralization assay would be suitable for the characterization of RBS binding antibodies.

As stated above, HA stalk-targeted monoclonal antibodies and HA head-targeted antibodies with no detectable HI titer may evoke potent ADCC activities both *in vitro* and *in vivo*. However, HI-positive antibodies that block RBS are commonly demonstrated not to induce robust ADCC activities [82,83,105]. This failure in inducing potent ADCC activity by HI-positive antibodies might be explained by a model proposed by Leon and colleagues [105]. In this model, two synapses between the effector cell and the virus-infected cells are essential for a robust ADCC response (Figure 3A,B). The binding between Fc and FcγRIIIa serves as the first synapse and the interaction between viral HA head and the SA on the effector cell forms the second synapse. These two synapses cooperatively reinforce the connection between the target cell and the effector cell, which mediate robust ADCC activity. The Fc-FcγRIIIa interaction alone is insufficient to induce potent ADCC activity and the interaction of HA to SA on the effector cell is required to induce potent ADCC function [82,83,105]. However, it remains obscure whether the SA-binding of HA activates a signaling pathway to induce ADCC or stabilizes the interaction between antibody and innate immune leukocytes [105]. HI-positive antibodies may compete with the SA on the effector cell in binding to the HA head domain, which as a result may impair the second synapse and hence lead to the reduced ADCC activity eventually (Figure 3C). It was reported that the *in vitro* ADCC activity induced by a stalk-reactive antibody is diminished after the addition of a HI-positive antibody [83,105]. The blocked or diminished ADCC activity may be due to the competitive role of neutralizing (HI-positive) antibody in binding to the HA. Vaccination can induce both neutralizing mAbs and nnAbs, future vaccine design should consider this delicate balance to achieve the optimal vaccine efficacy.

It is worth noting that neutralization and ADCC are not mutually exclusive. Antibodies with both anti-viral mechanisms have also been identified and characterized in recent studies, which demonstrated that ADCC activity could occur concurrently with a neutralization response [23,106]. Shen et al. reported that one HA head-targeted antibody, C12G6, can not only neutralize Yamagata, Victoria and earlier lineages of influenza B viruses, but also inhibits the membrane fusion, virus egress, and induces an ADCC response [106]. Notably, they also demonstrated that the potency of C12G6-mediated ADCC activity against different viruses followed an opposite trend to its HI activity, in which higher HI titer was found to be associated with relatively lower ADCC activity, and vice versa.

## 5. ADCC to Other Proteins in Influenza Virus

**Figure 4:**
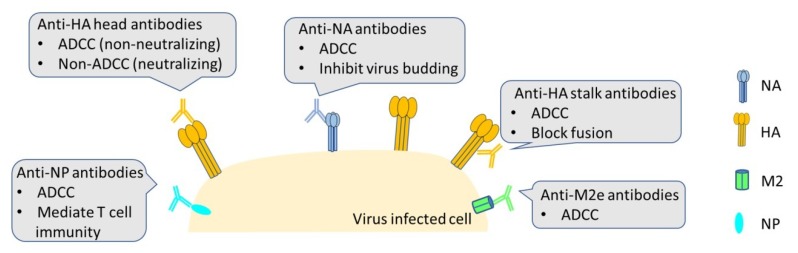
ADCC- mediating antibodies (ADCC-Abs) targeting different influenza antigens. HA, NA and M2 are the surface-exposed proteins and they are capable of inducing ADCC-Abs. Antibodies targeting the conserved epitopes in these proteins confer broad protection against divergent viruses. NP expressed on the surface of the influenza-infected cell serves as a promising target for ADCC-Abs.

Three influenza proteins are expressed on the viral membrane; HA, NA, and the Matrix 2 protein (M2) and are targets for ADCC-Abs (Figure 4). Although still critical in the viral life cycle, compared to HA, NA and M2 proteins have relatively lower expression on the viral surface and are not heavily targeted by the immune response. For this reason, NA and M2 are similarly less immunogenic and have a slower antigenic drift rate. This makes them attractive vaccine targets and recently more studies have been exploring this avenue [108,109,110].

The NA functions to cleave host SA receptors in the viral life cycle allowing the release of nascent viruses and NA-targeted mAbs have shown a protective effect in animal models by blocking this release mechanism [111]. Antibodies targeting NA are induced at lower level than antibodies targeting HA after vaccination, which may be due to the relatively less expression of NA on infected cells [112]. Some NA-targeted antibodies require Fc-FcγR interactions to induce protection [18,112]. DiLillio et al. reported that the broadly neutralizing NA-reactive antibodies, 3C05, can induce protective immunity *in vivo* in an FcγR-dependent manner [100]. Recently, Stadlbauer et al. showed that one monoclonal antibody generated from plasmablasts isolated from a human donor naturally infected with the H3N2 virus, designated 1G01, showed the broadest reactive spectrum to a variety of neuraminidases ranging from influenza A group 1 and group 2, to both influenza B virus lineages [113]. These antibodies exhibited multiple anti-viral activities including the blockade of virus particle release and ADCC. The combination of two anti-viral mechanisms contributed to the broad protection from lethal challenges *in vivo* by these antibodies targeting neuraminidase antigen. 

The M2 conducts the proton flux into the virion in the process of virus entry and equilibrates pH across the trans-Golgi network in the viral maturation stage [114,115]. Response against epitopes from the ectodomain of M2 (M2e) has been shown to induce protective immunity in mice [116]. M2e raises the hope for the universal vaccine because of its highly conserved sequence and antibodies targeting M2e with broad protective effects in animal models [117,118]. M2e vaccine candidates with universal vaccine potential are tested in clinical studies [109]. It is worth noting that mAbs targeting the M2e are believed to possess Fc-receptor dependent functions, like ADCC or ADCP [109,119,120,121]. Song et al. generated an M2e-targeted mAb (Z3G1) without detectable neutralizing activity. Interestingly, the Z3G1 antibody was found to be able to induce potent ADCC and CDC activities *in vitro* as well as demonstrated protective effect *in vivo* [122]. Bakkouri et al. further demonstrated the essential role of Fc receptors in anti-M2e antibody-induced protection in mice [117]. These studies indicate that M2e-based vaccines evoke cross-protective responses in an FcγR-dependent manner. Generation and characterization of mAbs against M2e can provide critical insights for the development of novel therapeutics against influenza virus infection. 

In addition to those proteins expressed on the viral surface, influenza nucleoprotein (NP) possesses highly conserved regions and is also detected on influenza virus-infected host cells surface [123,124]. This makes NP reachable by antibody and substantial efforts have been focused on the antiviral effects of anti-NP mAbs. The host immune response against NP may participate in multi-mechanisms against influenza infection. Cytotoxic T lymphocytes (CTL) can kill target cells via the recognition of NP antigen peptides presented by MHC-I molecules [125,126]. Berthoud et al. reported a T cell-inducing vaccine based on NP and M1, was safe and immunogenic in older adults [127]. These internal viral proteins have not only shown beneficial by inducing host CTL response, but also can serve as a target for protective mAbs. Jegaskanda et al. demonstrated that mAbs targeting M1 and NP can also elicit ADCC activity and these mAbs can be induced by influenza vaccination and natural infection in children [20,128]. However, the protective role of anti-NP ADCC-Abs has not been investigated in animal models. Since internal viral proteins are more conserved, these ADCC-Abs may be considered to be included in future universal vaccine design.

## 6. ADCC-Based Vaccine

To tackle the threat posed by influenza viruses, developing a universal vaccine that can induce broad immune response against both seasonal drift strains and emerging strains with pandemic potential is of heightening priority of influenza research. Considerable effort has been focused on the development of therapeutic agents and universal vaccines against influenza virus infection in recent decades. Protective antibodies that bind to conserved epitopes in the HA head, HA stalk, NA, NP, M2e have been isolated and characterized in the vaccine research. Most influenza vaccinology studies aim to induce a high titer of broad neutralizing antibodies (bnAbs), which are capable of conferring broad and durable protection against influenza virus infection, but nnAbs are less investigated. However, it is difficult to induce bnAbs through natural infection and the effectiveness of bnAbs was compromised after the emergence of mutant viruses. Therefore, nnAb has become an alternative option and numerous studies have shown the different protective mechanisms of nnAbs and bnAbs [16,20,129]. Although no neutralizing activities can be detected *in vitro*, nnAbs are competent to mediate Fc-dependent protection *in vivo* by eliciting Fc-dependent effector activities. The protective property exhibited by nnAb seems to make an equally potential candidate for universal vaccines and therapeutics against influenza infection. Therefore, Fc-dependent effector activities should be considered and recognized when we evaluate the protective effects of influenza therapeutic agents and vaccine candidates.

The ADCC activity serves as a protective mechanism of HA stalk-binding antibodies and ADCC-Abs also exhibit much greater cross-reactivity than classic neutralizing antibodies, defining a promising direction to universal influenza vaccines. Various animal models are used to assess the protective capacity of ADCC based vaccines, including mouse, ferret, and non-human primates. Florek et al. reported that the ADCC-Abs induced by vaccination are capable of inhibiting influenza virus shedding in cynomolgus macaques [130]. A study focusing on severe human infection cases in China and Australia suggested that antibodies from severe influenza survivors were more likely to exhibit potent ADCC activity, which highlights critical role of ADCC in combating influenza virus infections [131]. 

ADCC represents a promising strategy for vaccine development, but there are still several questions to be addressed. First, the pre-existing neutralizing antibodies may affect the efficacy of the ADCC-based vaccine. He et al reported that the HI-positive antibody can inhibit the potency of ADCC activity induced by HA stalk-specific antibodies by competing in HA binding [83]. Therefore, the efficacy of the ADCC-based vaccine may be affected by pre-existing immunity. Besides, the activation of pre-existing B cells may induce class switching from IgG to non-ADCC isotypes [132]. Second, vaccines that elicit antibodies targeting the M2 or the internal proteins may confer protection against severe influenza diseases, however, may not prevent viral infection since these antibodies mainly recognize proteins expressed on the infected cell membrane [99]. Third, whether sufficient ADCC-Abs can be induced by vaccination is unclear. The antigen needs to be carefully designed and inclusion of an adjuvant is also critical to protective outcome. Four, there are reports of antibody-dependent enhancement (ADE) in ADCC-mediating antibodies [71,133]. In these studies, ADCC epitopes were identified and these epitope-based vaccines were generated and tested in mice. The immunized animals with ADCC epitopes showed robust ADCC activities but had lower survival rates in comparison with the control group. In addition, it was shown that ADCC epitope-based vaccines might have side effects including extra tissue damage as seen in the lungs, which was caused by the high level of cytotoxic granules (perforin) induced by ADCC activation [71].

## 7. Strategies to Augment ADCC Activity

Augmenting ADCC activity is a novel research direction in cancer research. Target cell killing activities triggered by ADCC mAbs can be substantially increased by the addition of ADCC-promoting agents, which has attracted substantial attention in cancer research [134,135]. Means of augmentation of these responses in cancer research might shed light on influenza research as well. Different strategies for enhancing ADCC were developed and employed in clinical trials in recent studies.

Considering Fc- FcγRIII interaction is required to elicit ADCC activities, the potency of ADCC can be improved by strengthening this interaction. By selecting Fc variants with optimized Fc-FcγRIII affinity, antibodies can elicit a more robust activating signal to the effector cells [59]. Lazar et al. report that Fc variant S239D/I332E has the capacity to optimize Fc-FcγR interaction and enhance effector function both *in vitro* and *in vivo* [136].

Other strategies have been attempted to promote the secretion of cytokines by activating effector cells. Reovirus and toll-like receptor (TLR) agonists can increase NK-mediated ADCC by facilitating cytokines secretion [137]. TLR agonists can significantly enhance ADCC by increasing the percentage of activated NK cells. It has been reported that CpG-containing oligodeoxynucleotides (CpG ODN), TLR9 agonists, have the effect of promoting the cytokines secretion from an activated NK cell [138].

## 8. Conclusions and Future Directions

The protective efficacy of influenza vaccines largely depends on the stimulation of neutralizing antibodies. For a long period of time, vaccine efficacy has been evaluated by HI titers of neutralizing antibodies, which correlate fairly well with traditional views on vaccine-conferred protection. Recently it is becoming increasingly apparent that nnAbs also play a critical role in combating influenza infection even though *in vitro* antiviral activity is not detected in traditional assays [16,17,18]. Therefore, future evaluation of vaccine efficacy should take Fc-dependent antiviral functions into consideration to optimize vaccine efficacy. In this review, we have summarized multiple anti-viral mechanisms of monoclonal antibodies with ADCC function and reveal that ADCC can overlap substantially with other mechanisms to confer heterosubtypic protection against seasonal and pandemic influenza. The characterization of identified nnAbs constitutes a novel pathway that mayraise hope for the design of universal vaccines. However, there are still questions about ADCC-Abs need to be addressed.

(1) As mAbs mediated Fc-effector activities are epitope dependent, identifying epitopes that can elicit nnAbs will serve as a good start for the rational design of universal vaccines and therapeutics. Some studies show that ADCC-Abs recognize linear epitopes [70]. Arunkumar et al. demonstrated that antibodies recognizing conformational epitopes exhibit better protection compared to the antibodies targeting linear epitopes [17]. Further investigation should be focused on the elucidation of which epitope type can induce a more potent ADCC response and as a result provide better protection.
(2) ADCC-Abs may also exert immune pressure on the virus, resulting in escape mutants that evade antibody recognition similar to what is seen with traditional neutralizing antibodies. No influenza studies have reported ADCC-antibody escape mutants currently, but Chung et al. demonstrated the critical amino acids in the linear epitope could mutate to escape ADCC responses in HIV-1 [139]. Lee et al. summarized two potential mechanisms employed by HIV-1 to resist ADCC responses, i. by directly reducing envelope antigen expression on the infected cell surface and ii. by restricting the exposure of epitopes on the envelope to reduce antibody binding [56,140,141]. Similar or different scenarios may present in influenza, which may need further exploration in future studies.
(3) It remains unclear about the viral replication step(s) in which ADCC occurs. It is indicated that ADCC activity does not intervene in viral entry and fusion and there is a high possibility that ADCC may target the virus-infected cell, which has HA, or other virus protein expressed [142]. This should be better investigated.
(4) Designing antibodies with enhanced capacity to induce ADCC activities against influenza infection should be pursued vigorously. As stated above, new approaches are being developed to augment ADCC in cancer research. These studies in cancer research may provide valuable insights into influenza research towards developing better vaccines or therapeutics to improve influenza patient outcomes. Therefore, it would be beneficial to measure the ADCC potency threshold for protective immunity. However, it is worth noting that a more potent ADCC response may not guarantee better protection. Arunkumar et at. reported that two non-neutralizing antibodies, KL-BHA-9B9 and KL-BHA-4C10, exhibited uncorrelated ADCC activity with *in vivo* protection [17]. KL-BHA-9B9 had potent ADCC activity *in vitro* but poor protection *in vivo*, whereas KL-BHA-4C10 conferred strong protection *in vivo* but had almost undetectable ADCC *in vitro*. A better understanding of ADCC activation may facilitate the mechanistic elucidation of this phenomenon as well as other variabilities emerged in current assays.
(5) Although diverse assays have been developed to measure ADCC activity *in vitro*, it is still obscure to what extent these assays can correlate to the efficacy *in vivo*. It is challenging to predict ADCC-Ab efficacy in humans. The transgenic mouse model expresses human FcγRs may better evaluate the antibody therapeutic effect in humans. However, mice are not natural hosts of influenza and influenza viruses causing human epidemics or pandemics typically replicate inefficiently in this animal model unless adapted viruses are used. Non-human primate models such as rhesus macaques or cynomolgus macaques are used in ADCC-Abs research and research findings from this model collectively indicated that ADCC-Abs are associated with reduced shedding of influenza viruses [16,130]. Considering the different expression patterns of FcγRs in humans and non-human primate animals, it remains uncertain whether the correlation of treatment outcome can be expected in humans. In this regard, clinical studies regarding ADCC-Abs are instrumental to examine the potential role of ADCC-Abs in combating influenza virus infection in humans [143].
(6) It would be interesting and worthwhile to study the synergy between neutralizing antibody and non-neutralizing antibody *in vivo* since both classes of antibodies will be elicited after viral infection. The high complexity of each type of antibodies and the lack of an ideal animal model make this investigation relatively challenging. In some cases, the Fc-effector functions induced by nnAb can increase the potency of neutralizing antibodies [14]. In contrast, it is also revealed that Fc-effector functions may not play any role when there is a high dose of neutralizing antibodies [29]. One possible explanation for this is that the function elicited by neutralizing antibodies, such as viral entry blocking or fusion disruption, is potent and sufficient for *in vivo* protection alone.
(7) The interplay among ADCC-Abs targeting HA, NA, M2e and NP needs to be investigated. It is critical to identify what antibody combination patterns can induce synergistic protection to augment vaccine efficacy. It is reported that the NA-inhibiting antibody elicited weak ADCC response alone, but it may cooperate with antibodies targeting the HA stem to enhance their potency of ADCC activity [83]. NA inhibitors can enhance Fc-dependent functions induced by anti-stalk antibody, suggesting a therapeutic synergy between NA inhibitors and anti-stalk antibody in humans [144]. Deng et al. demonstrated that the vaccination with double-layered nanoparticles containing HA-stalk and M2e induced potent long-lasting immunity and full protection against divergent influenza A viruses challenge in mice, indicating that the combination of antibodies targeting HA stalk domain and M2e can improve the potency and breadth of vaccine-induced protection [145]. The cocktail of therapeutic antibodies may overcome the emergence of viral escape mutants [37]. Further studies on antibody or inhibitor interplay may guide the better design of future universal anti-influenza countermeasures with broad-spectrum protection via multiple mechanisms.
